# Supplementary material for: Crystallization and Temperature Driven Morphological Evolution of Bio-based Polyethylene Glycol-acrylic Rosin Polymer
Source: Polymers (Basel). 2019 Oct 15;11(10):1684. doi: 10.3390/polym11101684 (PMC6835602; doi:10.3390/polym11101684)
Supplement: Supplementary file 1 [file polymers-11-01684-s001.pdf]

Supplementary Information

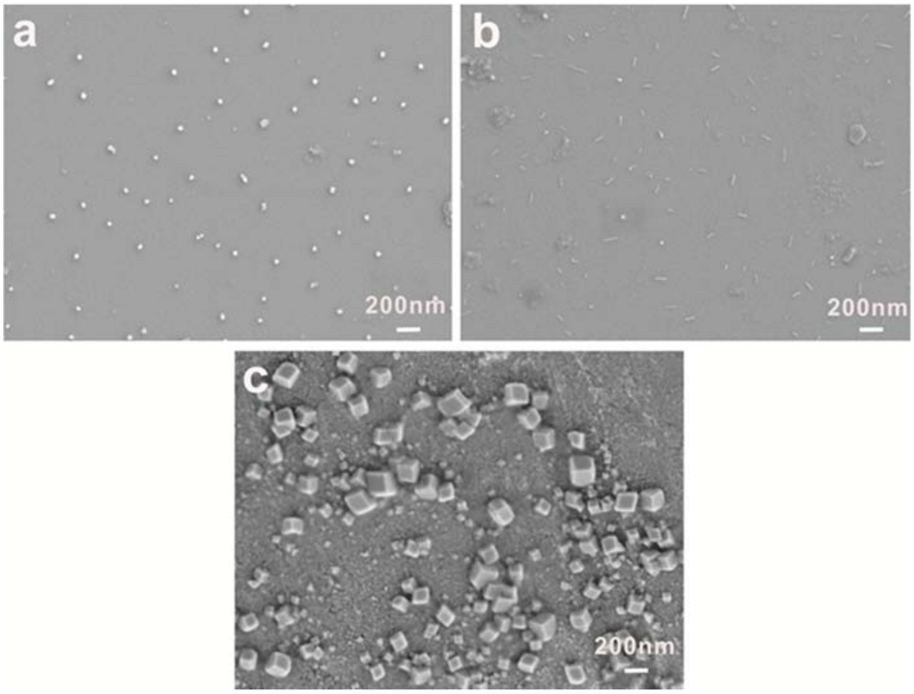

Figure S1. SEM images of 0.01 g L^{-1} PEG-acrylic rosin polymer at (a) 25 °C, (b) 85 °C and (c) 5 °C respectively.
